# Pediatric Anesthesia: The Essential Value of a Well-Supported Clinic

**DOI:** 10.3390/children12030263

**Published:** 2025-02-21

**Authors:** Alessandro Vittori, Marco Cascella

**Affiliations:** 1 Department of Anesthesia and Critical Care, ARCO ROMA, Ospedale Pediatrico Bambino Gesù IRCCS, 00165 Rome, Italy; 2 Anesthesia and Pain Medicine, Department of Medicine, Surgery and Dentistry “Scuola Medica Salernitana”, University of Salerno, 84081 Baronissi, Italy; mcascella@unisa.it

This Special Issue on pediatric anesthesia aims to showcase advanced research and expert perspectives that emphasize the crucial role of this specialty. The ten articles included in this issue explore innovative approaches and interventions designed to enhance perioperative care and optimize patient outcomes.

Pediatric anesthesia—understood as perioperative medicine, critical care medicine, and pain medicine—has established itself in recent decades as a subspecialty of anesthesia [1]. The international debate about the need for specific training of anesthesiologists regarding managing children, and even more so of newborns, is extremely lively [2]. This debate arises from the awareness that the anatomical–physiological peculiarities of the child and the neonate presuppose a solid basic knowledge on which a specific pediatric culture must be grafted [3].

Despite their limitations, important studies underline how an anesthesiologist’s experience has an important influence on perioperative complications [4]. On the other hand, quality cannot be defined only in terms of major complications, and not just confined to the field of perioperative medicine. There is a need for a high-quality standard even in critical care medicine and pain medicine, where the absence of complications does not mean quality is provided [5].

The main problem in pediatrics is that it is not possible to transfer the notions established about adults to children, and even more so for neonates [6]. The problem is twofold: the difficulty in conducting serious and rigorous scientific studies on these populations, and the difficulty in translating guidelines and studies concerning adults onto the pediatric population with a critical eye. The first problem afflicts all areas of anesthesia, but in particular chronic pain [7], where it becomes impossible to obtain certain scientific evidence in a reasonable period [8]. The second problem concerns the personal sphere of training. Reading and applying the literature without a critical spirit means becoming a technician and no longer a physician. This is valid in general, but even more so in the pediatric field. If the average anesthetist is supported by robust, serious, and rigorous scientific evidence, and by quality international guidelines, but must still read these documents with a critical eye, further effort is required of the pediatric anesthesiologist. Furthermore, critical reading is not enough; an important capacity for syncretism and mental flexibility is needed. As has been stated in an old movie, “*To know how to find you must first know how to hide*” [9]; similarly, in order to apply evidence effectively and safely, one needs to know how the scientific process works.

The main critical issue arises when, lacking supportive scientific evidence and, therefore, guidelines in the pediatric field, the literature regarding adults must be necessarily applied. Nevertheless, by adopting this literature without a critical spirit, we would be taking a leap back centuries, to the Platonian episteme, taking for granted and certain what in reality is not [10]. In practice, we would all skip that Hegelian path that ultimately leads us to the episteme, but with certain evidence and foundations [10]. All this has a notable practical implication, namely that what is our routine clinical practice cannot, and must not, be separated from research.

As expressed above, to know how to find, you need to know how to hide. Consequently, to apply scientific results correctly and carefully, we need to be good researchers. If this is true for any anesthetist, it is especially true for pediatric anesthetists. Among these, it is those who hold leadership roles who are called to make further efforts—leadership also in research. As is the case in a military career, in which the chain of command is well understood because to access a rank one has to go through all those below it, pediatric anesthesia leaders must have been able to demonstrate great research capabilities.

On these premises, the new challenge is not to limit oneself to training only in the field, but also, and above all first, to scientific training. Research, if conducted with rigor and seriousness, also represents a model of conduct for clinical care. Finally, we must not overlook the great networking capacity that research requires, which then allows a network of human and professional contact—also between structures—to be transferred to the healthcare field, which facilitates and speeds up the patient care process.

Therefore, the clinic working alone in pediatric anesthesia loses value.
